# Diverse functions of miR-373 in cancer

**DOI:** 10.1186/s12967-015-0523-z

**Published:** 2015-05-20

**Authors:** Furong Wei, Chuanhua Cao, Xiaoqun Xu, Junfu Wang

**Affiliations:** Institute of Basic Medicine, Shandong Academy of Medical Sciences, School of Medicine and Life Sciences, University of Jinan-Shandong Academy of Medical Sciences, 18877 Jingshi Road, 250062 Jinan, Shandong People’s Republic of China; Department of Oncology, Xiangyang Central Hospital, Affiliated Hospital of Hubei University of Arts and Science, Xiangyang, China

**Keywords:** microRNAs, miR-373, Cancer, Proliferation, Invasion, Oncogene

## Abstract

MicroRNAs (miRNAs) are small noncoding RNAs that regulate gene expression post-transcriptionally. They are involved in almost all cellular processes, and many have been described as potential oncogenes or tumor suppressors. MicroRNA-373 (miR-373), which was first identified as a human embryonic stem cell (ESC)-specific miRNA, is suggested to be implicated in the regulation of cell proliferation, apoptosis, senescence, migration and invasion, as well as DNA damage repair following hypoxia stress. Deregulation of miR-373 has been demonstrated in a number of cancers, whether it acts as an oncogene or a tumor suppressor, however, seems to be context dependent. In this review, we focus on the diverse functions of miR-373 and its implication in cancers.

## Introduction

MicroRNAs (miRNAs) are small noncoding RNAs approximately 22 nucleotides (nt) in length that are engaged in virtually all cellular processes [1, 2]. Generally, miRNAs negatively regulate gene expression by repressing translation or inducing degradation of target messenger RNAs (mRNAs). However, miRNAs can also positively regulate gene expression by modulating promoter activity or activating translation [3–5]. In mammals, miRNAs are predicted to control the activity of ~50 % of all protein-coding genes, and deregulated expression of miRNAs has been implicated in a variety of human diseases including cancer [2].

miRNA-373 (miR-373) was first identified as one of the human embryonic stem cell (ESC)-specific miRNAs [6]. Subsequently, it was validated as a potential novel oncogene with the evidence that it can permit proliferation and tumorigenesis of primary human cells harboring both oncogenic RAS and active wild-type p53 [7]. From that time forth, the functions of miR-373 in tumors as well as the potential targets of miR-373 have been an interested research field. miR-373 has been documented to suppress target mRNAs translation and/or degrade target mRNAs [7–28], besides, it can also induce target genes expression [4, 29–31]. Deregulation of miR-373 has been demonstrated in a number of cancers, while indisputable evidence has demonstrated that miR-373 is an oncogene [7, 8, 10, 11, 14, 15, 18–21, 32, 33], solid documents suggested its tumor suppressor character [13, 16, 22–24, 27, 30, 31, 34, 35]. The focus of this review is to highlight the diverse functions of miR-373 and its implication in cancer.

## Functions of miR-373

miR-373 is located in the chromosomal band 19q13.4. It belongs to miR-371-3 gene cluster that is transcribed into polycistronic primary transcript pri-miR-371-3. The pri-miR-371-3 is then processed into 3 pre-miRNAs (pre-miR371, pre-miR-372 and pre-miR-373), giving rise to four mature miRNAs (miR-371, miR-372, miR-373 and miR-373*) [6, 7]. miR-373 is one member of miR520/373 family, which consists of three different miRNA clusters possessing identical seed region, miR-302/367, miR-371/372/373 and miR-520 [8, 18, 22]. Therefore, when we discuss the functions of miR-373 here, we should bear in mind that other members of miR-520/373 family may possess the same functions.

**Fig. 1:**
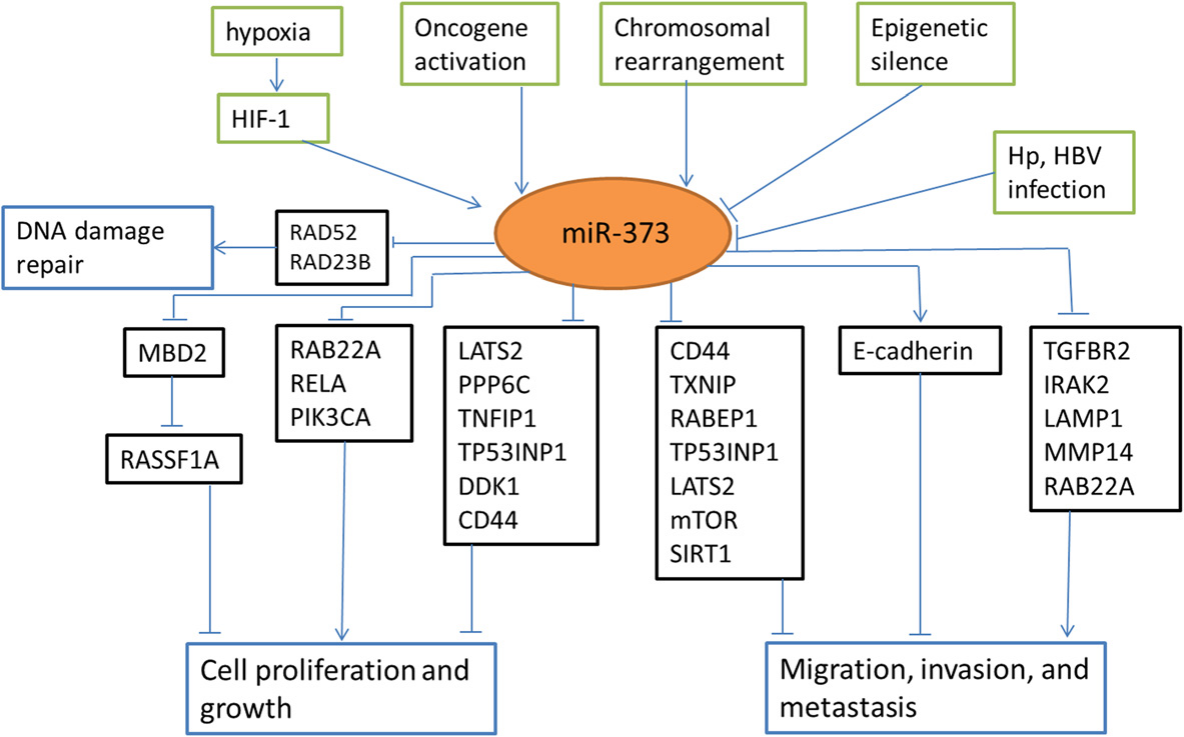
Regulation network of miR-373. Green boxes display upstream regulators of miR-373, black boxes contain representive functional targets of miR-373, and blue boxes imply the correspondingly involved cellular processes. Upstream factors lead to altered expression of miR-373. miR-373 exerts its functions by suppressing or promoting multiple target genes expression, and is involved in regulation of cell proliferation and growth, cell migration, invasion and metastasis, and DNA damage repair

miR-373 orchestrates its functions either by pairing to the 3’ untranslated regions (UTR) of specific mRNAs to post-transcriptionally down-regulate gene expression, or by binding to the promoters of target DNAs to up-regulate gene expression. Numerous target mRNAs and DNAs have been verified [4, 7–31], contributing to the potential roles of miR-373 in cellular processes (Table 1). So far, miR-373 has been elucidated to participate in the regulation of cell proliferation [10, 14, 16, 19–21, 23, 33], apoptosis [26, 27], senescence [7], mesendoderm differentiation [36], migration and invasion [8, 11, 18, 19, 23–25, 30, 31], it was also involved in hypoxia response as a hypoxia-induced miRNA, taking part in DNA damage repair [9] (Fig. 1).

**Table 1:** Verified direct targets of miR-373

Gene Symbol	Description	Regulation^a^	References
LATS2	large tumor tuppressor, homolog 2	↓	[7, 10, 12, 21]
CD44	CD44 molecule	↓	[8, 11, 21]
CDH1	cadherin 1, type 1, E-cadherin	↑	[4, 29–31]
CSDC2	cold shock domain containing C2, RNA binding	↑	[4]
RAD52	Rad52 DNA Repair and Recombination Protein	↓	[9]
RAD23B	RAD23 homolog B	↓	[9]
MBD2	methyl-CpG binding domain protein 2	↓	[13]
NFIB	nuclear factor I/B	↓	[17]
RAB22A	RAB22A, member RAS oncogene family	↓	[16, 24]
PPP6C	protein phosphatase 6, catalytic subunit	↓	[14]
TXNIP	thioredoxin interacting protein	↓	[15]
RABEP1	rabaptin, RAB GTPase binding effector protein 1	↓	[15]
RelA	v-rel avian reticuloendotheliosis viral oncogene homolog A	↓	[22, 27]
TGFBR2	transforming growth factor-beta type II receptor	↓	[19, 22]
mTOR	Mammalian Target of Rapamycin	↓	[18]
SIRT	sirtuin (silent mating type information regulation 2 homolog)	↓	[18]
DKK1	dickkopf homolog 1	↓	[19]
BTG1	B-cell translocation gene 1, anti-proliferative protein	↓	[19]
LEFTY1	left-right determination factor 1	↓	[19]
TNFAIP1	tumor necrosis factor, alpha-induced protein 1	↓	[20]
TP53INP1	tumor protein p53 inducible nuclear protein 1	↓	[21]
IRAK2	interleukin-1 receptor-associated kinase 2	↓	[23]
LAMP1	lysosomal-associated membrane protein 1	↓	[23]
MMP14	matrix metallopeptidase 14 (membrane-inserted)	↓	[25]
ER	estrogen receptor	↓	[26]
PIK3CA	phosphatidylinositol-4,5-bisphosphate 3-kinase, catalytic subunit alpha	↓	[27]
JAK1	Janus kinase 1	↓	[28]
IRF9	interferon regulatory factor 9	↓	[28]

^a^Expression regulation of target genes by miR-373: ↓ represents down-regulation, ↑ represents up-regulation

### miR-373 regulates cell growth

Cellular senescence is a state of irreversible cell cycle arrest, which limits the proliferative capacity of cells exposed to stress signals. Oncogene activation can induce cellular senescence, which stands as a natural barrier against tumorigenesis [37, 38]. However, by inactivation of tumor suppressors involved in senescence, such as P53 and RB, transformed cells overcome this barrier and reacquire proliferative capacity [39]. To identify miRNAs which cooperate with oncogenic RAS to overcome oncogene induced senescence, Voorhoeve et al. [7] developed a library of vectors expressing the majority of cloned human miRNAs, and identified miR-372 as well as miR-373 as an oncogene for the first time. While oncogenic stress leads to activation of P53 and induction of P21, which results in cell cycle arrest and senescence, the introduction of miR-372 or miR-373 prevents the inhibition of CDK by targeting tumor suppressor LATS2 directly, thereby permitting proliferation and tumorigenesis of transformed cells [7]. Subsequent investigations further reinforce the oncogenic potential of miR-373. In addition to LATS2 [10, 12, 21], many other tumor suppressors such as PPP6C [14], DKK1 [19], TNFAIP1 [20] and TP53INP1 [21] are also verified as direct targets of miR-373, implying that miR-373 promotes cell proliferation and tumor growth under certain conditions.

Notwithstanding the foregoing mentioned tumor promoting function of miR-373, a number of studies also presented the tumor suppressive potential of miR-373. miR-373 expression levels are down-regulated in a variety of cancers, including cholangiocarcinoma [13], colon cancer [16], pancreatic cancer [31], lung cancer [23] and ovarian cancer [24], overexpression of miR-373 could inhibit cell proliferation and tumor growth. Mechanism analysis revealed many putative oncogenes as the direct target of miR-373, including MBD2 [13], RAB22A [16], RelA and PIK3CA [27] (Table 1).

### miR-373 modulates cell migration and invasion

Invasion and metastasis represent the aggressiveness of cancer, predicting poor prognosis in patients. Exploring the underlying mechanism of cell migration and invasion is critical to improve our understanding of cancer metastasis and discover potential therapeutic targets [40]. Recent researches suggest that miRNAs are a set of important molecular regulators modulating cell migration and invasion [41–43].

miR-373 was first identified as a metastasis-promoting miRNA in breast cancer. Using a forward genetic screen, Huang et al. [8] transduced MCF-7 cells with a miRNA-expression library and performed transwell cell migration assay to assess their migration ability, identified miR-373 and miR-520c as metastasis-promoting miRNAs. CD44 was determined as a functional target of miR-373 and miR-520c, down-regulation of CD44 by miR-373 was responsible for the migration phenotype. Based on the metastasis-promoting function of miR-373 in MCF-7 cells, Yan et al. [15] implemented a quantitative proteomics to globally identify the genes regulated by miR-373. They evidenced that more than 30 proteins involved in cancer invasion and metastasis were found to be regulated by miR-373, among which TXNIP and RABEP1 were demonstrated to be the direct targets. In human fibrosaroma HT1080 cells, miR-373 and miR-520c can also promote migration. By targeting mTOR and SIRT1 directly, which are negative regulators of MMP9 expression, miR-373 and miR-520c up-regulates the expression level of MMP9, resulting in increased cell migration [18].

Paradoxically, miR-373 has been demonstrated to function also as a suppressor of cell migration and invasion. E-cadherin, a well-known regulator of migration and invasion [44], can be up-regulated by miR-373 [4, 30, 31]. Overexpression of miR-373 in A549 cells increased the expression level of E-cadherin, resulting in decreased migration [30]. The Hepatitis B × antigen (HBx), which is involved in HBV-related hepatocellular carcinoma (HCC), was shown to be able to down-regulate the expression miR-373 which correspondingly reduces E-cadherin expression, suggesting that HCC with low miR-373 expression level might be more invasive [29]. In addition to E-cadherin, TGFBR2 [22], RAB22A [24], MMP14 [25], IRAK2 and LAMP1 [23] were also identified as direct targets of miR-373 that contributing to its metastasis-inhibiting function.

### miR-373 takes part in hypoxic response

Hypoxia is a common feature of the tumor microenvironment and has been recognized as a culprit that confers hallmark capabilities to cancer cells [45]. Under hypoxic stress, cancer cells switch to glycolytic metabolism, develop self-sufficient growth signal, become resistant to apoptosis, acquire unlimited replication potential and genomic instability, evade immune attack, and induce angiogenesis and metastasis [46].

miRNAs have recently been shown to play an essential role in the regulation of the cellular response to hypoxia, which are termed as hypoxia-regulates miRNAs (HRMs) or HypoxamiRs [47]. Among the identified HRMs, only miR-210, the master HRM, shows consistent up-regulation under hypoxia in all cell types, other HRMs, including miR-373, response to hypoxia in a cell-specific way [48]. In cervical cancer cell line HeLa and breast cancer cell line MCF-7, miR-373 was up-regulated under hypoxia [9]. However, no significant up-regulation of miR-373 was detected in hypoxic prostate cancer cell lines [49].

miR-373, up-regulated under hypoxic conditions in certain cancer cells, has been demonstrated to play a role in hypoxic response. By reducing the expression of RAD52, which is an important factor in homology-dependent repair, and RAD23B, which is involved in nucleotide excision repair, miR-373 impairs the DNA damage repair, thereby resulting in genetic instability [9]. Whether hypoxia-induced miR-373 could regulate hypoxic cell growth or modulate hypoxic cell migration and invasion deserves further investigations.

Taken together, miR-373 functions paradoxically, it promotes cell growth, cell migration and invasion on one side, but on the other it yields the opposite effect. This phenomenon could be explained in an oversimplified way: because each miRNA could target hundreds of mRNAs or even DNAs, the ultimate effect of deregulation of one particular miRNA depends on the genetic characteristic of cancer cells as well as tumor microenvironment. Considering the heterogeneity of tumor, it is not hard to comprehend the unpredictable functions of miR-373.

## miR-373 and cancers: evidence from both cultured cancer cell lines and clinical samples

miR-373, either down-regulated or up-regulated, has been implicated in tumorigenesis of various types of tumors (Table 2). In addition to chromosomal rearrangements of 19q13.4 that result in overexpression of miR-373 [32], many upstream factors have been demonstrated to regulate the expression of miR-373. Hypoxia can induce the expression of miR-373 by activating transcription factors hypoxia inducible factor 1 (HIF-1) [9]. Activation of oncogenes, such as Myc [33], β-catenin [19], can also up-regulate miR-373 expression. As for down-regulation of miR-373, epigenetic silencing is the major reason [16, 23, 27, 35]. Meanwhile, infection of Helicobacter pylori (Hp) or Hepatitis B virus (HBV) can also inhibit miR-373 expression [12, 29]. Here, we dissect the functions of miR-373 from the perspective of specific cancer, highlighting its differential role in distinct cancer.

**Table 2:** Summaries of representative studies investigating the role of miR-373 in clinical samples

Tumor type	Sample type	miR-373 expression levels^a^	miR-373 function	References
TGCT	Primary tumor tissue	↑	oncogene	[7, 51]
TGCT	serum	↑	oncogene	[53, 54]
Breast cancer	Primary tumor tissue and metastatic lymph nodes	↑	oncogene	[8]
Breast cancer (ER^−^)	Primary tumor tissue	↓	TSG	[22]
Breast cancer	serum	↑	oncogene	[26, 59–61]
HB	Primary tumor tissue		oncogene	[33]
HCC	Primary tumor tissue	↑	oncogene	[14, 29, 33]
Hilar cholangiocarcinoma	Primary tumor tissue	↓	TSG	[13, 34, 35]
Gastric cancer	Primary tumor tissue	↑	oncogene	[20]
Esophageal cancer	Primary tumor tissue	↑	oncogene	[10]
Colon cancer	Primary tumor tissue	↓	TSG	[16]
Prostate cancer	Primary tumor tissue	↓	Oncogene	[11]
Thyroid adenoma	Primary tumor tissue	↑	Oncogene	[32]
Pancreatic cancer	Primary tumor tissue	↓	TSG	[31]
Lung cacner	Primary tumor tissue	↓	TSG	[23]

*TGCT* testicular germ cell tumor, *ER* ^*−*^ estrogen receptor negative, *TSG* tumor suppressor gene, *HB* hepatoblastoma, *HCC* hepatocellular carcinoma

^a^↑represents up-regulation, ↓represents down-regulation

### miR-373 and testicular germ cell tumors (TGCTs)

TGCTs are classified into two histopathological types: seminomas and non-seminomas. The non-seminomas comprise of embryonal carcinoma (EC, the stem cell component), teratocarcinoma (TC, somatic differentiation), and yolk sac tumor and choriocarcinoma (YS and CH, extra-embryonal tissues) [7, 50].

The oncogenic potential of miR-373 was first explored in TGCTs [7]. In cell lines originating from TGCTs, four out of seven expressed the miR-371-3 cluster, while in primary TGCTs tissue, most seminomas (28/32) and two thirds (14/21) of nonseminomas expressed miR-372/373. Another study detected the miRNA expression profiles of seminoma from formalin-fixed and paraffin-embedded (FFPE) surgical samples, presenting that miR-373 was up-regulated about 1530 fold in seminoma compared to normal testicular tissue [51]. Up-regulation of miR-373, leading to LATS2 suppression, dampens the p53 pathway which is intact in most TGCTs, and allows oncogenic mutations to accumulate, playing an essential role in tumorigenesis of TGCTs.

Unveiling the molecular mechanisms of tumorigenesis provides us plenty of useful information, facilitating our discovery of proper diagnostic and prognostic biomarkers as well as potential therapeutic targets. miRNAs have been recognized as important biomarkers in the management of tumors. In particular, circulating cell-free miRNAs, which exist in a variety of body fluids including blood with remarkable stability, have received much attention [52]. In the context of TGCT, serum miR-371-3 have been proposed as advantageous biomarkers for both diagnosis and follow-up of TGCTs, showing higher sensitivity than conventional biomarkers such as alpha fetal protein (AFP) and human chorionic gonadotropin (HCG) [53, 54]. However, more researches remain to be done to validate this claim.

### miR-373 and breast cancer

Breast cancer is a heterogeneous disease comprising four different subtypes: luminal A, luminal B, basal-like and human epidermal growth factor receptor 2 (HER2) positive [55]. These tumors exhibit diverse genetic alterations, present distinct gene expression profiles including miRNA expression profiles [56, 57]. Several studies have identified the deregulation of miRNAs in breast cancer, and a variety of miRNAs have been implicated in the regulation of breast cancer initiation and progression [58]. To date, functions of miR-373 in breast cancer remain controversial.

Based on results from in vitro and in vivo experiments, miR-373 can both promote and inhibit metastasis of breast cancer cells, functioning in a cell-specific way. In human breast cancer cell line MCF-7, which is characterized by non-migratory and non-metastatic phenotype, overexpression of miR-373 promoted cell migration and invasion. In contrast, in human breast cancer cell line MDA-MB-435, which expresses endogenous miR-373, down-regulation of miR-373 significantly weakened cancer cell migration and invasion [8]. However, in MDA-MB-231, which is an aggressively invasive ER^−^ breast cancer cell line, overexpression of miR-520c/373 blunted the invasive capacity of cancer cells [22].

Could the investigation of clinical samples shed light on functions of miR-373? The answer remains inconclusive. On one side, miR-373 was demonstrated to be positively correlated with higher metastatic phenotype [8], on the other, miR-520c/373 was shown to be negatively correlated with lymph node metastasis [22]. Both studies have small sample size, which might be insufficient to reach a consistent result in such a heterogeneous disease as breast cancer. Large scales of patient cohorts are needed to specialize the paradoxical functions of miR-373 in each subtype with different genetic context.

In parallel with the extensive investigations of circulating cell-free miRNAs as biomarkers, serum or plasma miR-373 was also explored as a potential biomarker in breast cancer [26, 59–61]. The expression levels of plasma miR-373 were found to be significantly higher in breast cancer patients with lymph node metastasis compared with those without lymph node metastasis, suggesting that plasma miR-373 has an ability to discriminate lymph node status of breast cancer [59]. In addition, serum miR-373 was found to be significantly higher in patients of breast cancer than healthy women, which indicated that miR-373 is a promising diagnostic biomarker. Serum miR-373 expression levels of HER2-negative breast cancer were higher than that of HER2-positive patients, showing us the probability that serum miR-373 might be utilized to estimate the HER2 status of the primary tumor [60]. Further study suggested that serum exosomal miR-373 is the predominant source of circulating miR-373, the level of serum exosomal miR-373 was associated with negative ER and PR status [28]. In HER2-positive patients, however, serum expression levels of miR-373 did not correlate with prognosis [61].

Nevertheless, there are visibly limited studies to speculate the potential role of miR-373 as a biomarker. Although a single miR-373 could be used as diagnostic and prognostic biomarker with considerable sensitivity and specificity, considering the innate heterogeneity of breast cancer, combination of a panel of relevant miRNAs is more reasonable and advisable. Therefore, more investigations are warranted.

### miR-373 and liver cancer

Since miR-373 was identified as an ESC-specific microRNA, its implication in cancer can be easily connected to embryonic carcinomas. In addition to TGCTs, the role of miR-371-3 cluster in hepatoblastomas (HBs), a rare embryonic neoplasm but a common pediatric liver cancer derived from liver progenitor cells, was also explored [33]. In a study conducted by Cairo et al. [33], they investigated the MYC-driven reprograming of miRNAs in HBs, identified two relevant miRNA cluster acting in an opposite way. miR-371-3 was the miRNA cluster that was up-regulated. A four-miR signature representative of these clusters, comprising miR-100, let-7a, miR-371, miR-373, was identified, which could be used to discriminate not only aggressive HBs but also invasive HCCs. Up-regulated miR-373 cooperating with other deregulated miRNAs, which confers stem cell-like characteristics to cancer cells, is a biomarker for poor prognosis.

Two other studies investigated the potential roles of miR-373 in HCCs [14, 29]. They both demonstrated the up-regulation of miR-373 in tumor tissues compared with non-tumor tissues. However, mechanism analysis from different perspectives showed us distinct functions of miR-373. Wu et al. [14] evidenced that miR-373 functions as an oncogene. By down-regulating PPP6C, miR-373 facilitates the proliferation of HCC cell lines HepG2 and QGY-7703. Arzumanyan et al. [29] underlined the impact of HBx in liver cancer, suggested that miR-373 expression levels were down-regulated by HBx in HepG2X cells and tissue sections from HBV infected patients, and suppressed expression of miR-373 in liver probably represents a more aggressive phenotype.

### miR-373 and other tumors

In addition to the foregoing mentioned tumors, miR-373 was widely investigated in many other tumors, including hilar chlangiocarcinoma [13, 34, 35], gastric cancer [20], esophageal cancer [10], colon cancer [16], prostate cancer [11], thyroid adenoma [32], pancreatic cancer [31] and lung cancer [23, 27] (Table 2).

In principle, miR-373 was considered to function as an oncogene or a tumor suppressor based on its up-regulated expression or down-regulated expression in tumors compared with non-tumor samples. Nevertheless, in prostate cancer [11], of which miR-373 expression levels were down-regulated in both tumor tissues and cancer cell lines, exogenous miR-373 did not arrest the growth of tumor but accelerate migration and invasion by impeding CD44 translation, suggesting miR-373 might act as an oncogene unexpectedly.

## Conclusion

Initially identified as an ESC-specific miRNA, miR-373 is one of the interested fields in recent years. While evidences from cancer cell lines suggested its oncogenic or tumor-suppressive functions, analysis of clinical tumor samples further confirmed that deregulation of miR-373 plays a critical role in tumorigenesis. However, considering the limited numbers of studies available as well as the bidirectional functions of miR-373 acting in a cell-specific way, more investigations are warranted. The following aspects deserve future attention. First, the upstream molecular regulators of miR-373, which are responsible for the altered expression of miR-373, such as HIF-1 [9], MYC [33] and MBD2 [34], need to be explored. Second, additional targets of miR-373 urge to be verified, which may reveal more crosslinks between miR-373 and classical tumor-related signaling pathways such as NF-κB, TGF-β [22], Wnt/β-catenin [19] and JAK/STAT [28]. This would promote us to better understand its pleiotropic functions in tumorigenesis. At last, with regard to utilize miR-373 as a biomarker either in diagnosis or prognosis of cancer, studies with large cohorts of patients are needed, and the combination of a variety of miRNAs including miR-373 may be more effective.
